# Does the Urothelium of Old Mice Regenerate after Chitosan Injury as Quickly as the Urothelium of Young Mice?

**DOI:** 10.3390/ijms21103502

**Published:** 2020-05-15

**Authors:** Eva Erzar, Mojca Kerec Kos, Katja Lakota, Peter Veranič, Andreja Erman

**Affiliations:** 1 Department of Biotechnology, Jožef Stefan Institute, 1000 Ljubljana, Slovenia; eva.erzar@gmail.com; 2 Faculty of Pharmacy, University of Ljubljana, 1000 Ljubljana, Slovenia; mojca.kerec-kos@ffa.uni-lj.si; 3 Department of Rheumatology, University Medical Centre Ljubljana, 1000 Ljubljana, Slovenia; katja.lakota@guest.arnes.si; 4 Institute of Cell Biology, Faculty of Medicine, University of Ljubljana, 1000 Ljubljana, Slovenia; peter.veranic@mf.uni-lj.si

**Keywords:** urothelium, young mice, old mice, chitosan, regeneration, inflammation

## Abstract

The aging of organisms leads to a decreased ability of tissue to regenerate after injury. The regeneration of the bladder urothelium after induced desquamation with biopolymer chitosan has been studied in young mice but not in old mice. Chitosan is a suitable inducer of urothelial desquamation because it is known to be non-toxic. We used chitosan for desquamation of urothelial cells in order to compare the dynamics of urothelial regeneration after injury between young and old mice. Our aim was to determine whether the urothelial function and structure of old mice is restored as fast as in young mice, and to evaluate the inflammatory response due to chitosan treatment. We discovered that the urothelial function restored comparably fast in both age groups and that the urothelium of young and old mice recovered within 5 days after injury, although the onset of proliferation and differentiation appeared later in old mice. Acute inflammation markers showed some differences in the inflammatory response in young versus old mice, but in both age groups, chitosan caused short-term acute inflammation. In conclusion, the restoration of urothelial function is not impaired in old mice, but the regeneration of the urothelial structure in old mice slightly lags behind the regeneration in young mice.

## 1. Introduction

The urinary bladder epithelium, called urothelium, is one of the most frequent targets of bacterial attacks on human tissues, especially in elderly women [1,2,3]. Our previous study discussed the most successful treatment of bacterial cystitis so far in an animal model using a combination of antibiotics and a biopolymer chitosan [4]. Although several studies have presented rapid regeneration of the urothelium in young animals [5,6,7,8], the regenerative process in old animals has not been described yet.

The main functions of the mammalian urinary bladder are collecting and controlling the voiding of urine. The luminal side of the mouse urinary bladder wall is covered by three-layered urothelium. It consists of undifferentiated basal cells at the basal lamina, partly differentiated intermediate cells, and terminally differentiated superficial cells called umbrella cells, which are in contact with the urine in the lumen of the urinary bladder [9]. The urothelium functions as a blood–urine permeability barrier, which is the tightest barrier in the mammalian organism, impermeable to urine compounds and potential pathogens in urine [10]. The blood–urine permeability barrier is maintained by superficial cells, which have a specialized apical plasma membrane with specific transmembrane proteins called uroplakins. The uroplakins are arranged in hexagonal crystals, which form plaques of thickened plasma membrane (12 nm), surrounded by interplaque regions of normally thick plasma membrane (7–10 nm) [11]. In the subapical region of the cytoplasm of umbrella cells, specific plaque-bearing vesicles called fusiform vesicles are concentrated, delivering uroplakins to the apical membrane, and a cytokeratin 20-network, which replaces actin filaments that disappear during urothelial cell differentiation [9,12,13,14]. An impermeable apical plasma membrane of umbrella cells together with well-developed tight junctions between them prevents the transcellular and paracellular passage of molecules through the urothelium [15,16].

Vertical differentiation of urothelial cells is a constant process, which is very slow under physiological conditions because the urothelium is a very stable tissue with low proliferative activity and umbrella cells have a lifespan of more than 6 months [9,17,18,19]. However, after bacterial infection or injury, which causes the disruption of tight junctions and desquamation of urothelial cells, proliferative activity and differentiation are rapidly induced, consequentially leading to rapid structural and functional regeneration [7]. For this reason, various chemicals have been used to induce urothelial injury and to study its regeneration or drug diffusion into the bladder stroma [5,13,20,21,22].

Unlike other chemical injury triggers, natural polysaccharide chitosan has been proven to be an effective and safe inducer of urothelial desquamation in ex vivo and in vivo conditions [7,8,23]. Depending on its concentration and the time of exposure, controlled desquamation of urothelial cells could be induced, followed by rapid functional and structural urothelial regeneration [7,8,23]. In addition, chitosan in combination with immunotherapy and antibiotics shows encouraging results in the treatment of some age-related diseases of the urinary bladder in animal models [4,24,25,26].

The regeneration of the bladder urothelium in young mice after induced desquamation with chitosan has already been studied under ex vivo and in vivo conditions [7,8]. However, we could not find any published data about urothelial regeneration in old mice, although it is established in general that old organisms are less able to restore damaged tissue and that a gradual decline in organ functioning appears with ageing in almost all organ systems [27,28,29]. Therefore, the purpose of our study was to compare the functional and structural regeneration of the urothelium between young and old mice after chitosan-induced injury. Moreover, we wanted to ascertain whether chitosan is really a non-toxic biopolymer that causes no inflammatory response, as mentioned in the literature [30,31,32,33]. For this reason, we analyzed the classic hallmarks of acute inflammation in the urinary bladder wall, characterized histologically by edema of the lamina propria and extravascular migration of neutrophil granulocytes. In addition, we analyzed the expression of serum amyloid A3 (*Saa3*), belonging to the SAA family of major acute inflammation proteins in humans and most other mammals, including mice, and cyclooxygenase 2 (*Cox-2*), the key enzyme for the formation of prostaglandins during inflammation [34,35,36]. By comparing all selected hallmarks of acute inflammation, we wanted to assess the severity of the inflammation response in young versus old mice.

## 2. Results

### 2.1. Restoration of Urothelial Function in Ex Vivo Experiments

**Figure 1:**
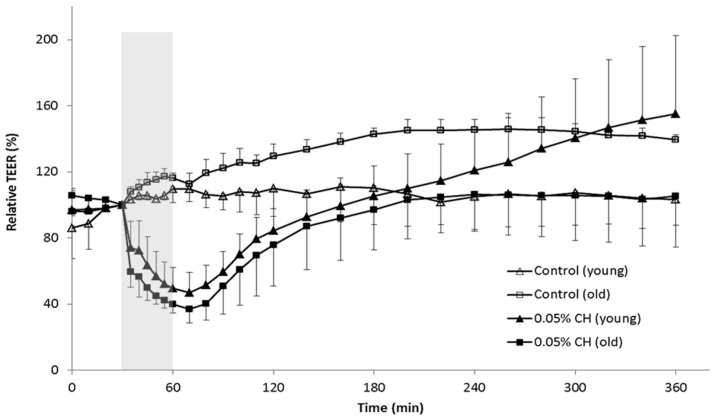
Transepithelial electrical resistance (TEER) values of urinary bladders of young and old mice measured in ex vivo experiments (mean and standard deviation). During the treatment period (grey shaded area), the urothelium was exposed to 0.05 % *w*/*v* dispersion of chitosan (CH) with a pH of 4.5 or phosphate buffer saline with a pH of 4.5 (control). Altogether, the isolated urinary bladders of 5 young and 5 old animals were used; for each age group, four urinary bladder halves in control experiments and six urinary bladder halves in the experiments with chitosan.

To evaluate the functional barrier integrity of the urothelium of young and old mice, transepithelial electrical resistance (TEER) measurements were performed in ex vivo conditions (Figure 1). In the first 30 min of the experiment (the equilibration period), the tissue was allowed to recover after the stress induced by the excision of the urinary bladder from the animal, and the TEER value at the end of this period was taken as the baseline value (i.e., 100%) to calculate the relative TEER. The exposure of the urothelium to phosphate-buffered saline (PBS) with a pH of 4.5 (control experiments) did not result in any decrease of TEER in either old or young animals. This is in line with our previous study on rat urinary bladders, in which the urothelium preserved its barrier function during the entire duration of the experiment, with no measurable functional or structural changes [8]. On the other hand, TEER values rapidly decreased during the exposure of the urothelium to 0.05% chitosan dispersion and reached the lowest level at the beginning of the regeneration period (at approximately 70 min). However, there was no significant difference in the relative TEER values between young and old mice at any time point between 35 and 70 min (*t* test, *p* = 0.111–0.138). During the regeneration period, when the chitosan dispersion was replaced with PBS (pH 7.4), TEER values constantly increased. The urothelium of old mice reached 100% TEER at approximately 140 min after the removal of the chitosan dispersion from the mucosal surface. In the case of the urothelium of young mice, the baseline value of TEER was reached 20 min earlier. Only in the last two time points of the experiment (at 340 and 360 min), the average TEER values of young mice were significantly higher in comparison to old mice (*t* test, *p* < 0.05). It can be concluded from the rapid fall of TEER values that the urothelium of young and old mice responded similarly to chitosan. Moreover, after chitosan removal, the recovery of the urothelial barrier function was comparable in both age groups of animals.

### 2.2. Results of In Vivo Experiments

#### 2.2.1. Morphological Evaluation of Urothelial Injury in Young and Old Mice

**Figure 2:**
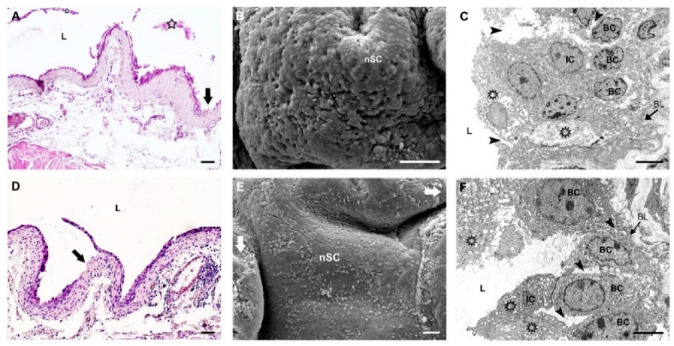
Representative micrographs of urothelial injury in young (**A**–**C**) and old (**D**–**F**) mice. The majority of the superficial cell layer (star frame in **A**) is peeled off and smaller intermediate cells as new superficial cells (nSC) are exposed on the urothelial surface. At some areas of the urothelium, desquamation extends into deeper cell layers, where dilated intercellular spaces (arrowheads in **C**,**F**), necrotic urothelial cells (asterisk frames in **C**,**F**), and exposed basal lamina are present (arrows in **A**,**D**,**E**). (**A**,**D**) Hematoxylin and eosin (H&E) staining; (**B**,**E**) Scanning electron microscopy; (**C**,**F**) Transmission electron microscopy. L-lumen of the urinary bladder, BL-basal lamina, IC-intermediate cells, BC-basal cells. Scale bars: 100 µm (**A**,**B**,**D**,**E**); 6 µm (**C**,**F**).

Two hours after chitosan treatment, the extent of urothelial injury was similar in young and old mice. In both age groups, the majority of superficial cells were desquamated and the urothelium was mostly two-layered with exposed intermediate cells as new superficial cells (Figure 2). These cells were smaller and at a lower cell differentiation stage than desquamated superficial cells. In some areas of the urothelium, intermediate and even basal urothelial cells desquamated, resulting in exposed basal lamina. Necrotic urothelial cells and increased intercellular spaces in deeper urothelial layers were also detected (Figure 2).

#### 2.2.2. Restoration of Urothelial Structure after Chitosan Treatment in Young Mice

**Figure 3:**
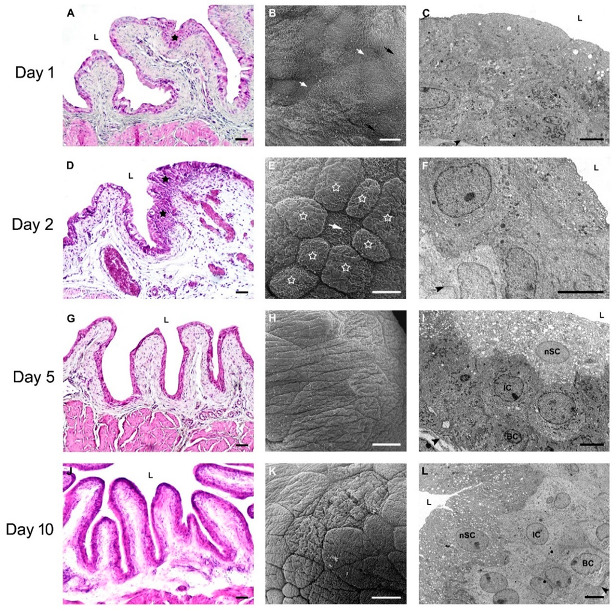
Representative micrographs of the urothelium during regeneration in young mice. On day 1 after chitosan treatment (**A**–**C**), the urothelium is mostly two-layered with some hyperplastic areas (black star in **A**) (**A**,**C**) and new superficial cells on the surface with microvilli (white arrows) or ropy ridges (black arrows) on the apical surface (**B**). On day 2 after chitosan treatment (**D**–**F**), the urothelium is three-layered with a few hyperplastic regions (black stars in **D**) (**D**,**F**). New superficial cells are small but at a higher cell differentiation stage with a specifically scalloped apical surface (star frames) or ropy ridges (white arrow) on the apical surface (**E**). On day 5 (**G**–**I**) and 10 (**J**–**L**) after chitosan treatment, the urothelium is continually three-layered (**G**,**I**,**J**,**L**). Superficial cells have a specific scalloped appearance of the apical plasma membrane, well-developed tight junctions (**H**,**K**), and numerous typical fusiform vesicles in the cytosol (**I**,**L**). (**A**,**D**,**G**,**J**) Hematoxylin and eosin (H&E) staining; (**B**,**E**,**H**,**K**) Scanning electron microscopy; (**C**,**F**,**I**,**L**) Transmission electron microscopy. L-lumen of the urinary bladder, BC-basal cells, IC-intermediate cells, nSC-new superficial cells. Black arrowheads show basal lamina. Scale bars: 100 µm (**A**,**D**,**G**,**J**); 10 µm (**B**,**E**); 20 µm (**H**,**K**); 6 µm (**C**,**F**,**I**,**L**).

One day after chitosan treatment, the urothelium of young mice was predominantly two-layered due to preceding desquamation of superficial cells and had a few hyperplastic areas (Figure 3A,C). Tight junctions and other intercellular junctions were well developed, and cell desquamation was no longer present at this time point. The luminal surface was composed of new superficial cells of various sizes and at different stages of cell differentiation from cells at a lower stage of differentiation with microvilli to more differentiated cells with ropy ridges on their apical surface (Figure 3B). Two days after chitosan treatment, the urothelium was three-layered again with some hyperplastic areas (Figure 3D). The urothelial surface was composed of new superficial cells, heterogeneous in both the cell size and the appearance of the apical plasma membrane (Figure 3E). The majority of new superficial cells were at a higher differentiation stage than on the first day after chitosan treatment. These cells were still small, with ropy ridges or the specific scalloped appearance of an apical plasma membrane, and rare fusiform vesicles in the apical cytoplasm (Figure 3F). On days 5 and 10 of the regeneration period, the entire urothelium was three-layered (Figure 3G,J). The urothelial surface was composed of highly differentiated superficial cells with well-developed tight junctions between them (Figure 3H,K). All superficial cells had a typically scalloped apical plasma membrane (Figure 3H,K) and numerous fusiform vesicles in the entire cytoplasm, which are characteristics of terminally differentiated urothelial cells (Figure 3I,L).

#### 2.2.3. Restoration of Urothelial Structure after Chitosan Treatment in Old Mice

**Figure 4:**
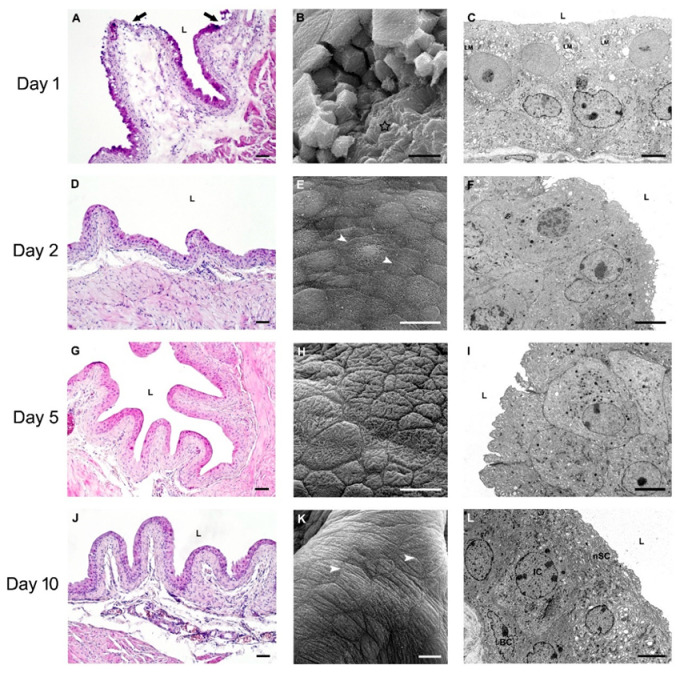
Representative micrographs of the urothelium during regeneration in old mice. On day 1 after chitosan treatment (**A**–**C**), the urothelium is mostly two-layered (**A**,**C**) with some areas of completely denuded urothelium, where the basal lamina is exposed (arrow in **A**, star frame in **B**). New superficial cells have numerous lamellar bodies (LM) in the cytoplasm (**C**). On day 2 after chitosan treatment (**D**–**F**), the urothelium is predominantly three-layered (**D**,**F**) with some hyperplastic regions and well-developed tight junctions (arrowheads) between superficial cells (**E**). The urothelial luminal surface is composed of a heterogeneous population of superficial cells, which differ in size and structure of the apical surface, but cells with ropy ridges are in the majority (**E**). On day 5 after chitosan treatment (**G**–**I**), the urothelium is mostly three-layered (**G**,**I**). Superficial cells are of various sizes and differentiation stages, but cells with a typically scalloped apical plasma membrane are dominant (**H**). On day 10 after chitosan treatment (**J**–**L**), the urothelium is continually tree-layered (**J**,**L**) with a homogeneous population of large superficial cells having a typically scalloped apical plasma membrane, well-developed tight junctions (arrowheads in **K**), and numerous fusiform vesicles in the cytoplasm (**L**). (**A**,**D**,**G**,**J**) Hematoxylin and eosin (H&E) staining; (**B**,**E**,**H**,**K**) Scanning electron microscopy; (**C**,**F**,**I**,**L**) Transmission electron microscopy. L-lumen of the urinary bladder, BC-basal cells, IC-intermediate cells, nSC-new superficial cells. Scale bars: 100 µm (**A**,**D**,**G**,**J**); 10 µm (**B**); 20 µm (**E**,**H**,**K**); 6 µm (**C**,**F**,**I**,**L**).

One day after chitosan treatment, the urothelium of old mice was mostly two-layered due to desquamation. In some areas, intense desquamation of urothelial cells was still present (Figure 4A,B), extending even to the basal lamina. The majority of the urothelial surface was composed of new superficial cells of various sizes and at different stages of cell differentiation, from cells at a low differentiation stage with microvilli to cells at a higher differentiation stage with ropy ridges of plasma membrane. In their cytosol, numerous lamellar bodies and mitochondria with electron-dense inclusions were detected (Figure 4C). Two days after chitosan treatment, the urothelium was two- to three-layered with some hyperplastic areas (Figure 4D). Superficial cells were of different sizes and with different appearances of the apical plasma membrane, revealing that they were at various stages of cell differentiation. Cells with microvilli were no longer present at this stage (Figure 4E). Relatively small numbers of fusiform vesicles were observed in the apical cytoplasm of new superficial cells (Figure 4F). Five days after chitosan treatment, some hyperplastic regions were still present in the predominantly three-layered urothelium, whereas 10 days after chitosan treatment, the urothelium did not have any hyperplastic areas (Figure 4G,J). At both time points, the majority of superficial cells had a typically scalloped apical plasma membrane, and among them, individual smaller cells with ropy ridges of apical plasma membrane were present (Figure 4H,K). In the cytoplasm of new superficial cells, numerous fusiform vesicles were found (Figure 4I,L).

#### 2.2.4. Proliferative and Apoptotic Activity of the Urothelium after Chitosan Treatment in Young and Old Mice

**Figure 5:**
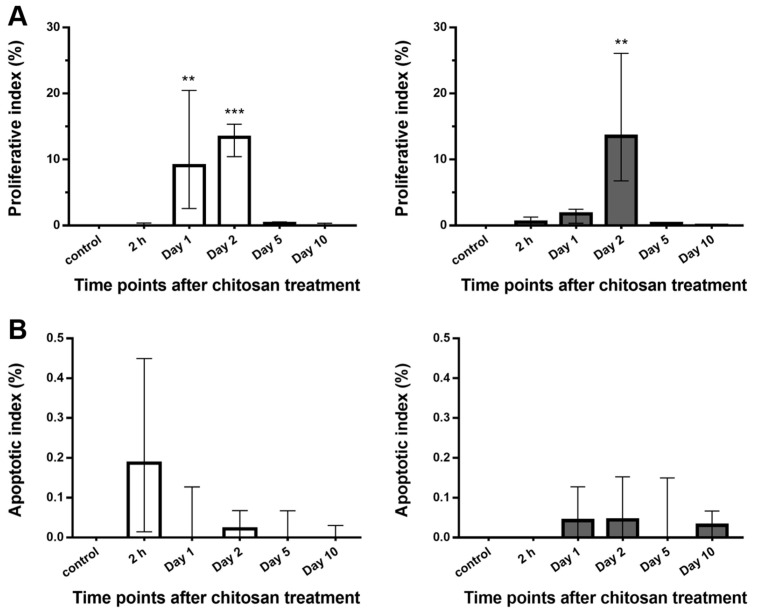
(**A**) Proliferative activity of the urothelium in young mice (white columns) is significantly increased on day 1 and 2 after chitosan treatment and in old mice (grey columns) on day 2 after chitosan treatment. Two tissue sections from each of 3 animals per time point. (**B**) Apoptotic activity of the urothelium is increased only 2 h after chitosan treatment in young mice (white columns) while in the urothelium of old mice (grey columns), apoptotic activity is low at all selected time-points. Two tissue sections from each of 2 animals per time point. Median ± interquartile range (IQR). ** *p* < 0.01, *** *p* < 0.001.

In young mice, high proliferative activity of urothelial cells was confirmed on the first and second days after chitosan treatment, and in old mice on the second day after chitosan treatment. At the other time points during the regeneration, the proliferative activity of the urothelium of both age groups was very low. Ki-67-positive cells were observed in all cellular layers of the urothelium. In untreated animals, no Ki-67-positive cells were found in either age group (Figure 5A).

In the urothelium of young mice, increased apoptotic activity was only observed 2 h after chitosan treatment, whereas at other time points during regeneration, apoptotic activity was weak. In the urothelium of old mice, apoptotic activity was not detected before the first day after injury and it was low during the entire regeneration period. Active caspase-3-positive cells were observed in the intermediate and basal cell layer of the urothelium of both age groups. In the urothelium of young and old control mice, no active caspase-3-positive cells were found (Figure 5B).

#### 2.2.5. Urothelial Differentiation after Chitosan Treatment in Young and Old Mice

##### Expression of Uroplakins

**Figure 6:**
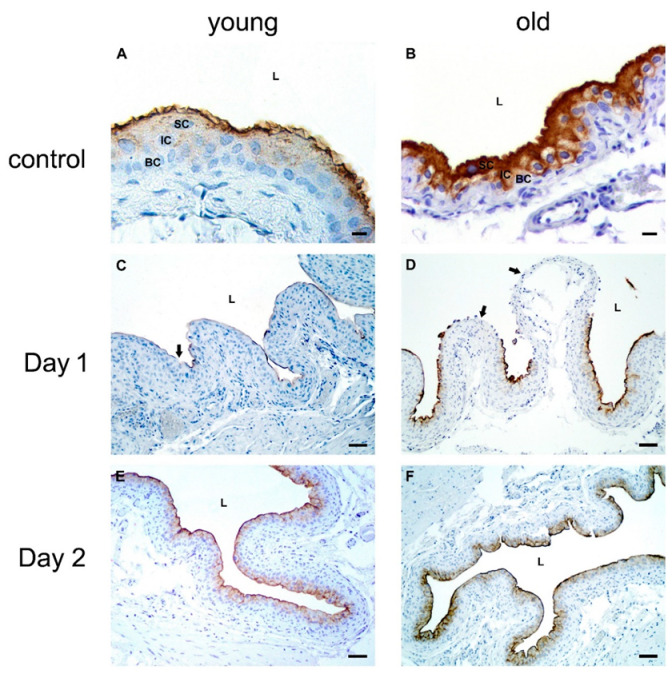
Representative micrographs of immunohistochemical detection of uroplakins in the urothelium of young (**A**,**C**,**E**) and old (**B**,**D**,**F**) mice. In the urothelium of control young (**A**) and old (**B**) mice, all superficial cells (SCs) show intense positive anti-uroplakin immunoreaction (brown colour) in their apical plasma membrane and apical cytoplasm. On day 1 after chitosan treatment, uroplakins are expressed weakly in new superficial urothelial cells of young and old mice (**C**,**D**), while in some denuded areas of the urothelium, no uroplakin expression is present (arrows). On day 2 after chitosan treatment, all superficial urothelial cells express uroplakins (**E**,**F**). Cell nuclei are stained blue with hematoxylin. L-lumen of the urinary bladder. IC-intermediate cells, BC-basal cells. Scale bars: 10 µm (**A**,**B**); 100 µm (**C**–**F**).

In the urothelium of young and old control mice, uroplakins as specific proteins of the apical plasma membrane and membrane of fusiform vesicles were strongly expressed in all superficial cells and weakly in intermediate urothelial cells (Figure 6A,B). On the first day after chitosan treatment, weak expression of uroplakins was observed in the majority of new superficial urothelial cells in young and old mice, whereas in some areas where desquamation took place into deeper urothelial layers, no immunohistochemical reaction against uroplakins was detected (Figure 6C,D). From the second day after chitosan treatment onward, a strong immunohistochemical reaction against uroplakins was observed again in all new superficial and intermediate urothelial cells in both age groups of mice (Figure 6E,F).

##### Expression of Cytokeratin 20

**Figure 7:**
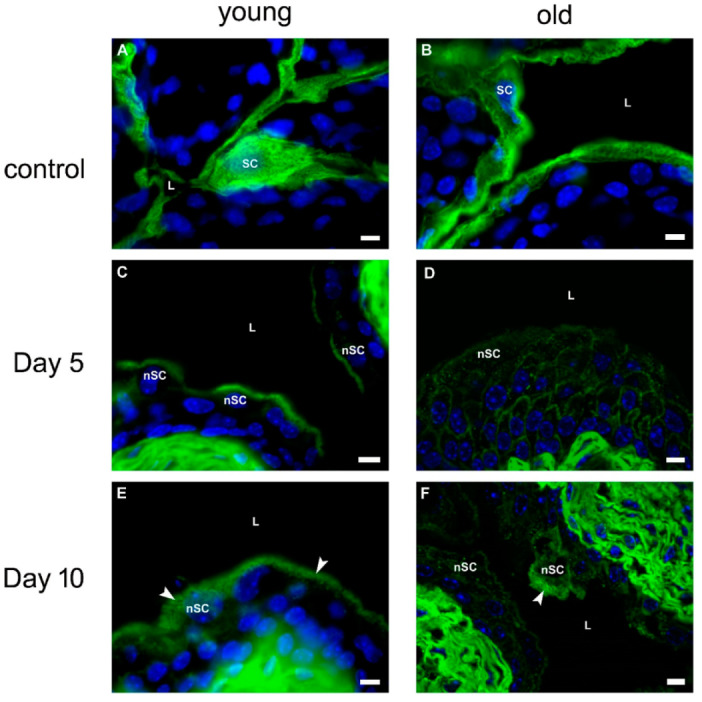
Representative micrographs of immunofluorescence detection of cytokeratin 20 (CK20) (green fluorescence) in the urothelium of young (**A**,**C**,**E**) and old (**B**,**D**,**F**) mice. In the urothelium of control young (**A**) and old (**B**) mice, the cytokeratin 20 (CK20) network is present in all superficial cells (SCs). On day 5 after chitosan treatment, the CK20 network appears in some new superficial cells (nSCs) of young mice (**C**), while in old mice, rare superficial cells express CK20 but are not organized into the network (**D**). On day 10 after chitosan treatment, all new superficial cells (nSCs) in young mice have a thin layer of the CK20 network in the apical cytoplasm (arrowheads) (**E**), while in old mice, only individual new superficial cells (nSCs) possess the CK20 network (arrowhead) (**F**). Cell nuclei are stained with 4′,6-diamidino-2-phenylindole (DAPI) (blue fluorescence). L-lumen of the urinary bladder. Scale bars: 10 µm (all images).

In the urothelium of young and old control mice, all superficial cells had a typical network of cytokeratin 20 in their subapical cytoplasm (Figure 7A,B). On the first and second days after chitosan treatment, no immunohistochemical reaction against cytokeratin 20 (CK20) was found in new superficial cells in the urothelium of either age groups. It was not until day 5 after chitosan treatment that we observed the established CK20 network only in individual new superficial urothelial cells in young mice. In the urothelium of old mice, we detected only a punctate immunohistochemical reaction in a few superficial cells and no CK20 network in their apical cytoplasm (Figure 7C,D). Ten days after chitosan treatment, in the urothelium of young mice, we observed the CK20 network in all superficial urothelial cells (Figure 7E), while in the urothelium of the old mice, the CK20 network was present only in individual superficial cells (Figure 7F).

#### 2.2.6. Acute Inflammation in the Bladder Wall after Chitosan Treatment in Young and Old Mice

##### Histological Evaluation of the Bladder Edema

**Figure 8:**
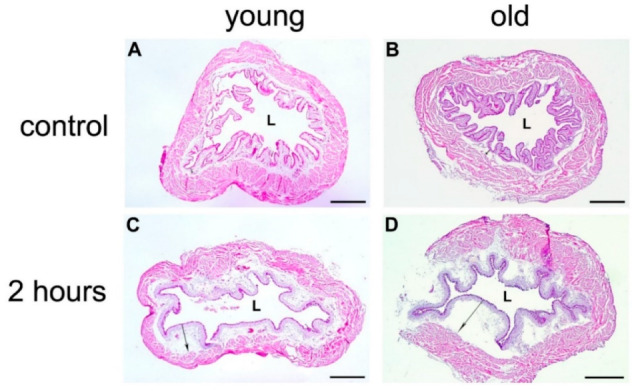
Representative images of urinary bladder cross-sections of the control young and old mice with marked normally thick lamina propria (arrow) (**A**,**B**) and 2 h after chitosan treatment with marked thickened lamina propria due to edema (arrow) (**C**,**D**). Hematoxylin and eosin (H&E) staining. L-lumen of the urinary bladder. Scale bars: 0.5 mm (all images).

**Figure 9:**
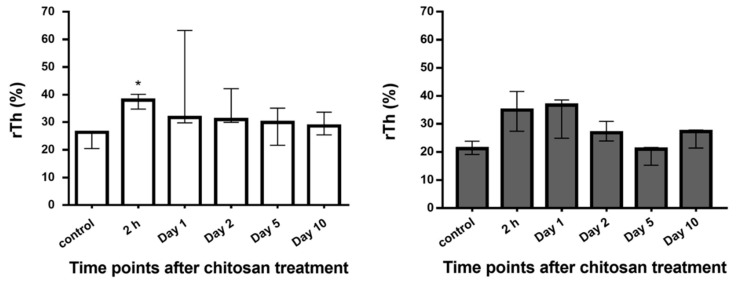
The relative thickness of lamina propria (rTh) in the urinary bladder wall of young mice (white columns) is significantly enlarged 2 h after chitosan treatment, while the increase is not significant in old mice (grey columns). Three tissue sections from one animal per each time point. Median ± interquartile range (IQR). * *p* < 0.05.

Two hours after chitosan treatment, the lamina propria of the urinary bladder was already noticeably thicker in comparison to control mice in both age groups as evidenced by histological images (Figure 8). The results obtained by analysis of the histological images shows that in young and old mice, the median relative thickness of the lamina propria already reached the maximum value 2 h after chitosan treatment (Figure 9). At subsequent time points of the regeneration period, the median relative thickness of the lamina propria gradually decreased, and on day 10, it was only slightly higher in comparison to the controls in both age groups (Figure 9).

##### Neutrophil Infiltration

**Figure 10:**
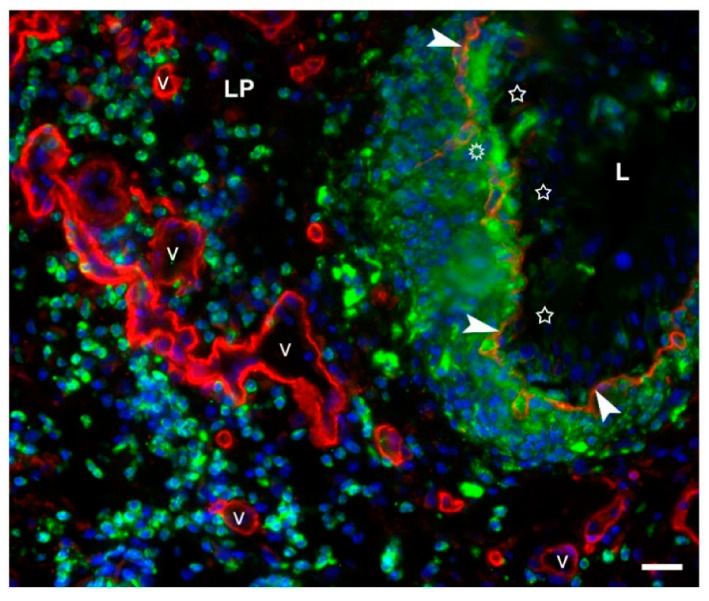
Representative micrograph of immunofluorescent detection of neutrophils in the urinary bladder wall of young mice on day 2 after chitosan treatment. Numerous extravascular and tissue-invading neutrophils (green fluorescence) are present in the lamina propria (LP) and even in the lumen (L) of the bladder. Collagen IV (red fluorescence) is immunolabeled to mark the basal lamina under the urothelium (arrowheads) and endothelium of blood vessels (v). Note that the basal lamina (asterisk frame) under the urothelium and the urothelium (star frames) are damaged at some places. Cell nuclei are stained with 4′6-diamidino-2-phenylindole (DAPI) (blue fluorescence). Scale bar: 20 µm.

In young and old mice, extravascular migration of neutrophils in the urinary bladder wall was the most intense from 2 h to 2 days after chitosan treatment, as is evident from the highest total scores at these time points in the scoring scheme (Table 1). In young mice on days 1 and 2 of the regeneration period, we observed an uncountable number of immunohistochemically marked neutrophils extended through the entire urinary bladder wall (Figure 10). In old mice, there was a peak of neutrophil infiltration on day 1 after chitosan treatment, as seen in Table 1. In both age groups, on days 5 and 10 after chitosan treatment, only a few neutrophils were found in the extravascular tissue of the urinary bladder wall (Table 1).

###### Expression of Cyclooxygenase 2 (*Cox-2*) and Serum Amyloid A3 (*Saa3*)

**Figure 11:**
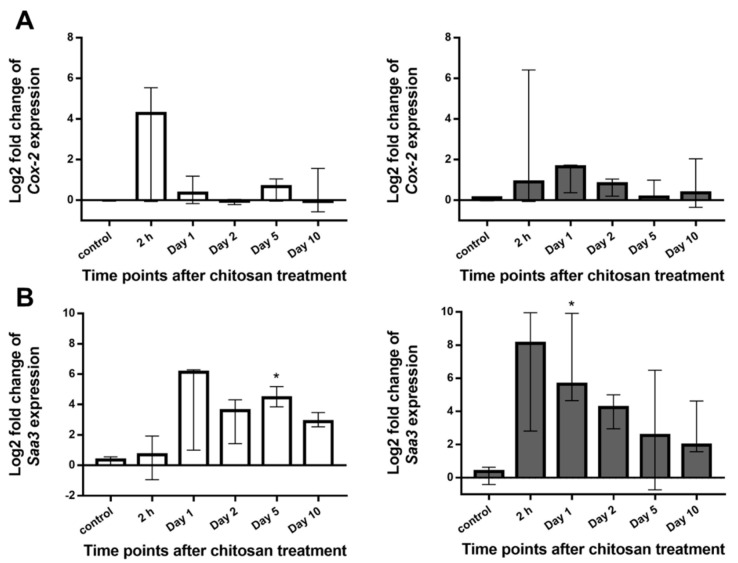
(**A**) Quantification of cyclooxygenase 2 (*Cox-2*) mRNA expression in the urinary bladder of young (white columns) and old (grey columns) mice. (**B**) Quantification of serum amyloid A3 (*Saa3*) mRNA expression in the urinary bladder of young (white columns) and old (grey columns) mice. In the period after chitosan treatment, median *Saa3* expression levels were higher in comparison to the control mice of both age groups. Three animals per each time point. Median ± interquartile range (IQR). * *p* < 0.05.

The peak of cyclooxygenase 2 (*Cox-2*) expression in the urinary bladder wall was obtained 2 h after chitosan treatment in young cyclooxygenase 2 mice and 1 day after chitosan treatment in old mice (Figure 11A). Two hours after chitosan treatment, the median *Cox-2* expression level was elevated 20-fold change in young mice, whereas in old mice, median *Cox-2* expression levels at all time points after chitosan treatment were not drastically elevated and slightly increased expression was obtained even in control mice (Figure 11A).

In contrast to *Cox-2* expression, serum amyloid A3 (*Saa3*) expression levels were elevated at all time points in young and old mice. On the first day after chitosan treatment, the median *Saa3* expression was increased more than 60-fold change in young mice and also significantly increased by 50-fold change in old mice (Figure 11B). *Saa3* was significantly upregulated 5 days after chitosan treatment in young mice, whereas in old mice, *Saa3* expression gradually decreased after day 1 (Figure 11B).

## 3. Discussion

It is generally accepted that the regenerative ability of damaged tissues decreases with age in organs, such as the heart, skeletal muscles, nervous system, and liver [27,28,29]. However, the regenerative ability of the bladder urothelium in old animals after injury has not been studied yet. The course of urothelial regeneration in young mice after chitosan-induced injury has already been studied [7,8]. An ex vivo study has shown that urothelial exposure to 0.005% chitosan caused a 60% decrease in TEER, the exposure of undifferentiated urothelial cells to the luminal surface, and leaky tight junctions [8]. In the in vivo experiment of our previous study, intravesical application of chitosan dispersion into mice of the same concentration resulted in complete removal of only the superficial cell layer within 20 min of treatment [7]. Furthermore, due to its effect on the urothelium, chitosan has been shown to be an auxiliary therapeutic agent in young mice with bacterial cystitis for removing the infected urothelial cells [4]. In view of the aforementioned, it was of great interest to us to determine whether the functional and structural regeneration of the urothelium after chitosan-induced injury in old mice is as fast as in young mice. For this reason, we compared the dynamics of the regeneration between young and old mice under ex vivo and in vivo conditions. In addition, to determine whether chitosan induces inflammation in the urinary bladder wall and to compare the extent of the inflammation response in both age groups, we studied the classic hallmarks of acute inflammation as well as the expression of *Saa3* and *Cox-2* [34,35].

In this study, TEER measurements in the ex vivo experiments showed a rapid drop of TEER when the urothelium was exposed to chitosan, and the decrease in TEER did not significantly differ between young and old mice. The control experiments showed that the decrease in TEER cannot be attributed to the pH of the buffer, in which chitosan was dissolved, or the experimental conditions used, which is in accordance with our previous study [8]. It is known from the literature that chitosan increases the permeability of the urothelium due to the disruption of tight junctions, which induces desquamation of urothelial cells [7,8,23,37]. Consequently, measured TEER is decreased, but after the removal of chitosan, TEER values gradually increase to the initial values due to tissue regeneration. We expected slower restoration of the urothelial barrier function in old mice compared to young mice or even that the urothelium of old mice would not fully recover its function until the end of the experiment. Surprisingly, the rate of recovery was comparable in both age groups of animals, and the baseline value of TEER (the value at the end of the equilibration period) was reached at approximately the same time. This rapid restoration of the urothelial permeability barrier in old mice confirms the fact that a functional blood–urine barrier is of vital importance for all organisms at any age and that the formation of an impermeable apical plasma membrane and the formation of tight junctions are leading events in urothelial regeneration after injury.

Morphological examination of urothelial injury 2 h after chitosan treatment revealed a similar extent of injury in both age groups of mice, considering the range of cell desquamation in the width and the depth of urothelial tissue. At that time point, the urothelium was mostly two-layered because chitosan disrupted tight junctions between umbrella cells and caused their elimination and necrosis of underlying cells. Necrotic urothelial cells were detected in both age groups, which is in accordance with our previously published results [7].

During regeneration, proliferation and cell differentiation as the two main processes restore cell deficiency in damaged tissue. Therefore, we also focused on these two processes to acquire and compare data about the regeneration dynamics of the urothelium in young and old mice, which has not yet been elucidated in old mice. For urothelial tissue, the ultrastructure of the apical plasma membrane and proteins associated with differentiation, such as cytokeratins and uroplakins, are well-known and useful differentiation markers. They make it possible to distinguish between different stages of urothelial cell differentiation and thus indirectly assess the regeneration grade of the urothelium after injury at a particular time point of regeneration [7,38,39]. In addition to the aforementioned markers, many other differentiation markers exist that help tracking the regeneration dynamics of the urothelium, such as keratin 5, keratin 14, and P63, respectively. By these markers, together with CD44 and CD49f, it is even possible to identify basal cells that have properties of stem cells and have crucial roles in urothelial regeneration. Since there is no data about stem cells in the urothelium of old organisms, it would be of great interest to analyze whether differences in the stem cell population exist between young and old mice, but this is beyond the scope of our study [40].

It has been known for a long time that the normal urothelium of adult mice is a very stable tissue with low proliferative activity [17]. This study did not detect any Ki-67-positive urothelial cells, neither in control young mice nor in control old mice, and so it proved that the normal urothelium of old mice is also proliferatively quiescent tissue, which has not been previously published. In addition, we showed for the first time that the urothelium of old mice also has a capacity for a rapid proliferative response after acute damage, as has been known for a long time in young mice [41,42,43,44]. Altogether, based on the differentiation markers and proliferation activity that we examined, it can be concluded that urothelial regeneration in old mice is slightly delayed in comparison to young mice.

One day after chitosan treatment, in contrast to young mice, the urothelium of old mice was still injured even to the basal lamina in some areas, and without hyperplastic regions, which coincides with a low proliferative and apoptotic index at this time point. These results indicate that chitosan had a more toxic effect on the urothelium of old mice because the injury lasts longer than in young mice, causing a delay in proliferative activity and consequently also in cell differentiation. However, 2 days after chitosan treatment, the urothelium of both age groups of mice was mostly again three-layered, and in some areas still hyperplastic, which coincided with the proliferative indices that were the highest at this time point of the regeneration. On days 5 and 10 after chitosan treatment, the urothelium of young and old mice was three-layered with low proliferative activity. All new superficial cells were at a higher stage of cell differentiation than on day 2. At these two time points, the difference in the urothelial differentiation stage between young and old mice was best seen by the final differentiation marker, the CK20 network [13], which appeared in individual superficial urothelial cells in young mice on day 5, while in old mice, the CK20 network did not appear until day 10. To sum up, the urothelial differentiation of old mice lagged behind urothelial differentiation in young mice but only at the subcellular structural level.

Ultrastructural analysis of urothelial tissue revealed no major differences between young and old mice, with the exception of the presence of numerous lamellar bodies and mitochondria with electron-dense inclusions found only in the urothelial cells of old mice until day 10 after chitosan treatment. We have already reported about dense inclusions in mitochondria in aging mice, and lamellar bodies have also been detected with transmission electron microscopy (TEM) in different cell types in normal or pathological conditions [45,46]. Their formation may be triggered by various therapeutic agents [47,48]. The appearance of lamellar bodies in cells where they are not physiologically present is associated with disorders in lipid metabolism or impaired autophagy [46]. We therefore assume that the formation of lamellar bodies in the urothelium was induced by a disruption of the plasma membrane or damaged mitochondrial membranes in response to chitosan-induced damage of urothelial cells. An increased number of lamellar bodies in old mice indicates slower degradation of damaged cell material by autophagy because diminished autophagic activity is one of the characteristics of the aging process [49,50,51]. We can summarize that the modified ultrastructure of the urothelial cells in the old mouse urothelium reflects a different response of these cells to the chitosan action in comparison to the urothelial cells of young mice. Moreover, Lui and colleagues [52] observed similar ultrastructural changes of the mitochondria in urothelial cells of the adult mice mutants with downregulated peroxisome proliferator-activated receptor gamma (*Pparg*). They showed that *Pparg* is critical for mitochondrial function and urothelial differentiation in vivo. Since similar abnormalities of mitochondria were observed in urothelial cells of mutant mice with downregulated *Pparg* and urothelial cells of old mice, we can speculate that *Pparg* expression in the urothelium of old mice is lower than in adult mice.

Many studies suggest chitosan as a non-toxic or low-toxic natural polymer [30,31,32,33]. However, by analyzing the classical hallmarks of acute inflammation, such as neutrophil infiltration and edema, we confirmed the development of acute inflammation 2 h after chitosan-induced injury, which lasted until the second day after chitosan treatment in young and old mice. In young mice, the expression of enzyme cyclooxygenase 2 (*Cox-2*) also showed the maximum at 2 h after chitosan treatment, whereas in old mice, we noticed slightly increased levels of *Cox-2* on day 1 after chitosan treatment. Taking into account these results and slightly elevated levels of *Cox-2* in old control mice, we may assume that mild chronic inflammation constantly persists in the urinary bladder wall of old animals, so the inflammatory response to noxious stimuli is not so intense as in young animals. Interestingly, major differences were recorded between young and old animals in the expression of serum amyloid A3 (*Saa3*). Because mice do not have acute-phase C-reactive protein (CRP), the detection of elevated concentrations of another acute-phase protein called serum amyloid A (SAA) is one of the key methods for detecting and confirming acute inflammation in mouse tissues [34]. It is established that SAA is a chemoattractant responsible for the induction of tissue infiltration of monocytes and polymorphonuclear leukocytes and that, within just a few hours after the onset of acute inflammation, the serum concentrations of SAA increase up to 1000-fold [53]. The SAA family consists of four isoforms in mice, which are differentially expressed during inflammation. One of our previous studies showed transiently elevated levels of *Saa1/2* in response to urinary tract infection (UTI) and also *Saa3* levels after intraperitoneally introduced uropathogenic *Escherichia coli* in mice [54]. The results of this study showed a significantly increased level of *Saa3* expression 5 days after chitosan treatment in young mice, and in old mice already 1 day after chitosan treatment. It seems that the inflammation response in old mice is faster and more intense at the gene level than at the histological level. So far, these are the first data about *Saa3* expression as part of the acute inflammatory response in the urinary bladder of young versus old mice. The results of this study undoubtedly prove that chitosan triggers short-term acute inflammation in the bladder wall of both young and old mice.

In conclusion, one of the main findings of this study is that the recovery of the permeability barrier as the main functional characteristic of the urothelium is not impaired in old mice. However, the structural restoration of the urothelium in old mice lags behind the structural restoration of the urothelium in young mice. Our results offer new data on urothelial regeneration in mice that might be translated to humans, also pointing to the potential use of chitosan as an auxiliary therapeutic agent in the treatment of cystitis in elderly people because both the regeneration and local acute inflammation are relatively short term and similarly intense in young and old animals.

## 4. Materials and Methods

### 4.1. Animals

All experiments were performed on female C57BL/6JOLaHsd young mice (8–16 weeks old) and old mice (24 months old; Harlan, Udine, Italy). The mice were housed (Medical Experimental Center, Ljubljana, Slovenia) in open-barred cages with bedding (Lignocel ¾; Altromin, Rosenberg, Germany) and enrichment material (paper towels) under controlled conditions of room temperature (22–24 °C), humidity (45–65%), and light (12 h/12 h light-dark cycle; 7 a.m.–7 p.m. light) and they had unlimited access to laboratory food (standard diet for rodents; Mucerola, Italy) and water.

For ex vivo experiments, the isolated urinary bladders of 5 young and 5 old mice were used, and for in vivo experiments, urinary bladders from 35 young and 20 old mice were isolated. Mice were sacrificed by CO_2_ inhalation and their urinary bladders were obtained immediately thereafter. All procedures involving mice were approved by the National Medical Ethics Committee and the Administration of the Republic of Slovenia for Food Safety, Veterinary, and Plant Protection (Permit number U34401-4/2016/8). Animal care and treatment were in accordance with Slovenian and international legislation and policy (Directive 2010/63/EU) on the protection of animals used for scientific purposes and also in accordance with the principles of the 3Rs for more humane animal research.

### 4.2. Ex Vivo Experiments

**Scheme 1:**
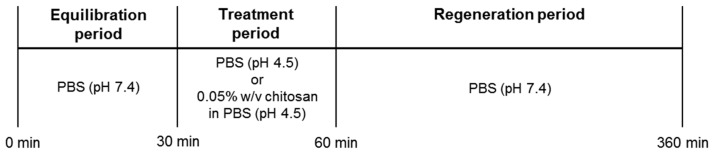
Schematic outline of ex vivo experiments. PBS-phosphate buffer saline.

After excision, urinary bladders were placed in Dulbecco’s modified Eagle’s cell culture medium with 1 mg/L glucose and sodium bicarbonate (DMEM; Sigma-Aldrich Chemie, Steinheim, Germany). The corpus of the urinary bladder was halved and then the tissue was placed onto an insert with a circular aperture (2 mm diameter; 0.031 cm² area). The insert was placed between the two EasyMount^®^ half-chambers of a diffusion chamber (Physiologic Instruments, San Diego, CA, USA). No pharmacological agents were used to relax the bladder smooth muscles prior to mounting the tissue to prevent their effects on epithelial integrity. During the entire experiment, the temperature was maintained at 37 °C and the incubation solutions were constantly oxygenated and stirred by bubbling with gas (95% O_2_, 5% CO_2_). The serosal side of the tissue was incubated in cell culture medium throughout the experiment. The urothelium was first exposed for 30 min to phosphate-buffered saline (PBS) with a pH of 7.4 (equilibration period). In the treatment period, the urothelium was exposed for 30 min either to PBS with a pH of 4.5 (control experiments) or to 0.05% *w*/*v* dispersion of chitosan (88.8% deacetylated chitosan hydrochloride, Kraeber & Co. GmbH, Ellerbek, Germany), prepared in PBS with a pH of 4.5. In the regeneration period, the urothelium was again exposed to PBS with a pH of 7.4 for 300 min (Scheme 1).

The electrophysiological parameters were measured with a multi-channel voltage-current clamp (model VCC MC6, Physiologic Instruments, San Diego, CA, USA). To measure the potential difference and passing current, the diffusion chambers were equipped with two pairs of Ag/AgCl electrodes connected to the chambers via 3M KCl/3.5% agar bridges. The experiments were performed under open-circuit conditions with the current set to zero. In this way, the natural transepithelial potential difference could be observed. Electrical readings were recorded every 10 min in the equilibration period, every 5 min in the treatment period, and every 10 min in the regeneration period until 120 min, and afterward every 20 min. For each measurement, the potential difference (PD) value was clamped to 20 mV and the necessary current was recorded. Electrical resistance was determined according to Ohm’s law. Afterward, the fluid resistance measured before mounting the tissue in the diffusion chambers was subtracted and the net electrical resistance of the urothelium was multiplied by the exposed tissue area (0.031 cm²) to obtain the transepithelial electrical resistance (TEER). To ensure the comparability of TEER, measurements are presented as a percentage of the TEER value at the end of the equilibration period of the particular tissue.

### 4.3. In Vivo Experiments

**Scheme 2:**
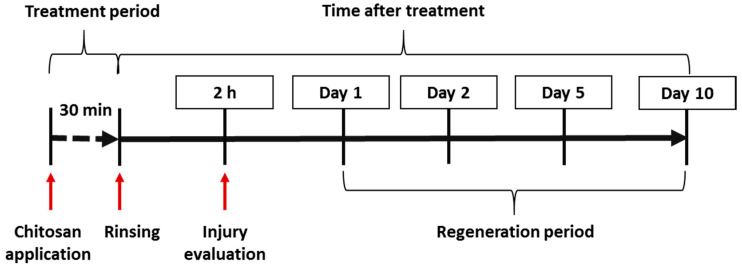
Schematic outline of in vivo experiments. Broken line represents the treatment period with chitosan and continuous line represents the period after chitosan treatment with marked time-points of sacrificing animals.

The mice were anesthetized with ketamine HCl (100 mg/kg intraperitoneally) and xylazine (10 mg/kg intraperitoneally), they were placed in the dorsal position, and a polyethylene catheter with a 0.28-mm inner diameter (Intramedic, Becton Dickinson, Franklin Lakes, NJ, USA) was inserted into the bladder through the urethra and sheathed over a 30-G needle connected to a 1-mL injection syringe. The bladder of each animal was emptied by mild manual pressure on the abdomen and 0.5% *w*/*v* dispersion of chitosan (88.8% deacetylated chitosan hydrochloride, Kraeber & Co. GmbH, Ellerbek, Germany) prepared in PBS (pH 4.5) was instilled for 30 min via transurethral catheterization. After that time, chitosan dispersion was washed out with a sterile physiological saline solution (0.9% NaCl) and the catheter and injection syringe were removed from the bladder. All infusions were performed gradually and at a slow rate to avoid injury or vesicoureteral reflux. At certain time points (2 h, day 1, day 2, day 5, and day 10) after intravesical application of chitosan, the mice were euthanized, and their urinary bladders were aseptically removed and processed for further analysis (Scheme 2).

### 4.4. Preparation of Paraffin Sections and Cryosections

For the preparation of paraffin sections, the excised urinary bladders from in vivo experiments were halved and fixed in 10% neutral buffered formalin for 24 h at 4 °C. After fixation, the bladder halves were rinsed overnight in PBS (pH 7.2–7.4), dehydrated, and embedded in paraffin. The tissue was sectioned into 5-µm-thick sections and stained with hematoxylin and eosin (H&E) or immunolabeled.

For the preparation of cryosections, the excised urinary bladders were halved and fixed in 3% paraformaldehyde in PBS buffer for 2 h at 4 °C, rinsed in 30% sucrose overnight at 4 °C, embedded in Tissue Freezing Medium (Leica, Wetzlar, Germany), frozen, and cut in a cryostat chamber CM3000 (Leica, Wetzlar, Germany) into 5-µm-thick cryosections. The cryosections were air-dried for at least 3 h before immunolabeling.

#### 4.4.1. Immunolabeling on Paraffin Sections

Paraffin sections were deparaffinized in xylol and rehydrated in ethanol series. Heat-induced epitope retrieval was performed in a microwave oven for 10 min at 600 W in citrate buffer (pH 6.0). After cooling, the permeabilization in 0.4% Triton X-100 (10 min; room temperature (RT)) was followed in the case of immunofluorescence labeling of neutrophils and active caspase 3 and in the case of immunolabeling of uroplakins and Ki-67 before the standard 3,3′-diaminobenzidine (DAB) development procedure. Later, tissue sections were incubated in 3% H_2_O_2_ in methanol to inactivate cell peroxidases (15 min; RT), and then in blocking solution (5% fetal calf serum (FCS) and 1% bovine serum albumin (BSA) in PBS) at 37 °C for 1 h to prevent unspecific binding of the antibodies. Overnight incubation of sections with appropriate primary antibodies (diluted in 1% BSA in PBS) at 4 °C (all listed in Table 2) followed. After rinsing in PBS, sections were incubated with appropriate secondary antibodies diluted in 1% BSA in PBS at 37 °C for 1 h (Table 2). Proper negative controls were also performed, in which primary antibodies were replaced with 1% BSA in PBS. For the immunolabeling of uroplakins and Ki-67 antigen, the standard DAB (Sigma-Aldrich, Steinheim am Albuch, Germany) development procedure was performed followed by the staining of sections with Mayer’s hematoxylin, dehydration, and mounting in DePeX (Serva Electrophoresis, Heidelberg, Germany). For the immunofluorescence labeling of neutrophils, Ki-67, and active caspase 3, after incubation in secondary antibody and rinsing in PBS, the sections were mounted in Vectashield antibleaching mounting medium with 4′,6-diamidino-2-phenylindole (DAPI) (Vector Laboratories, Burlingame, CA, USA) for DNA staining. Samples were observed and images obtained with a Nikon Eclipse E200 bright-field microscope (Nikon, Amsterdam, The Netherlands) and AxioImager Z1 fluorescence microscope (Carl Zeiss, Oberkochen, Germany).

#### 4.4.2. Immunofluorescence Labeling on Cryosections

Cryosections were first rinsed in PBS (15 min); in some cases, sections were additionally fixed in cold acetone (when immunolabeled with the following primary antibodies: anti-cytokeratin 20, anti-neutrophils, and anti-collagen IV) or permeabilized in 4% Triton X-100 in PBS (when immunolabeled with anti-active caspase 3 primary antibodies). After rinsing in PBS (15 min), sections were incubated in blocking solution (PBS buffer containing 5% FCS and 1% BSA) for 1 h at 37 °C and then incubated overnight with primary antibodies (diluted in 1% BSA in PBS) at 4 °C (Table 3). After rinsing in PBS (15 min), cryosections were incubated with appropriate secondary antibodies diluted in 1% BSA in PBS (Table 3) for 1 h at 37 °C and then again rinsed in PBS. The samples were mounted in Vectashield antibleaching mounting medium with DAPI (Vector Laboratories, Burlingame, CA, USA) for DNA staining. Proper negative controls were also performed, in which primary antibodies were replaced with 1% BSA in PBS. Samples were observed and images obtained with an AxioImager Z1 fluorescence microscope (Carl Zeiss, Oberkochen, Germany).

### 4.5. Analysis of Proliferative and Apoptotic Activity

For each time point after chitosan treatment, we took two tissue sections immunolabeled against Ki-67 from each of three animals (n = 6) and two tissue sections immunolabeled against active caspase 3 from each of two animals (n = 4). For the analysis of proliferative or apoptotic activity, cells with a positive immunohistochemical reaction against Ki-67 or active caspase 3 and all urothelial cells per tissue section were counted with a Nikon Eclipse E200 bright-field microscope (Nikon, Amsterdam, The Netherlands) and AxioImager Z1 fluorescence microscope (Carl Zeiss, Oberkochen, Germany). From the obtained values per each time point after chitosan treatment, we calculated the proliferative index (PI) and apoptotic index (AI) according to the equation:

(1) $$\mathrm{PI}\;\mathrm{or}\;\mathrm{AI}\;=\frac{\mathrm{Number}\;\mathrm{of}\;\mathrm{Ki}67\;\mathrm{or}\;\mathrm{caspase}\;3-\mathrm{positive}\;\mathrm{cells}}{\mathrm{Number}\;\mathrm{of}\;\mathrm{all}\;\mathrm{urothelial}\;\mathrm{cells}\;\mathrm{per}\;\mathrm{tissue}\;\sec\mathrm{tion}}\times 10$$

### 4.6. Transmission Electron Microscopy (TEM)

Excised urinary bladders were cut into small pieces and fixed in a mixture of 4.5% paraformaldehyde and 2% glutaraldehyde in 0.2 M cacodylate buffer (pH 7.3) for 3 h at 4 °C. Overnight rinsing in 0.33 M sucrose in 0.2 M cacodylate buffer at 4 °C was followed by post-fixation with 1% osmium tetroxide (OsO_4_) in 0.1 M cacodylate buffer for 1 h in the dark at room temperature. After post-fixation, the tissue samples were rinsed with distilled water and contrasted for 1 h in 2% uranyl acetate in the dark at room temperature and again rinsed with distillated water. After dehydration in an ethanol series, tissue samples were first impregnated with propylene oxide then left overnight in a mixture (1:1) of propylene oxide and Epon (Serva, Electrophoresis), and a day later tissue samples were embedded in Epon. Epon semi-thin sections (1 µm thick) were stained with 1% toluidine blue and 2% borate in distilled water for 20 s and observed with a Nikon Eclipse TE bright-field microscope (Nikon, Amsterdam, The Netherlands). Ultrathin sections were contrasted with uranyl acetate and lead citrate and examined at 80 kV with a Philips CM100 transmission electron microscope (Philips, Eindhoven, The Netherlands).

### 4.7. Scanning Electron Microscopy (SEM)

Excised urinary bladders were cut into flat pieces and fixed for 3 h at 4 °C in a mixture of 2% paraformaldehyde and 2% glutaraldehyde in 0.2 M cacodylate buffer (pH 7.4). The tissue samples were rinsed in 0.2 M cacodylate buffer overnight and post-fixed in 2% OsO_4_ in the same buffer (1:1) for 1 h in the dark at room temperature. Post-fixation was followed by dehydration in an acetone series gradient, after which specimens were critical-point dried, attached to aluminum holders, sputter-coated with gold, and examined at 30 kV with a Tescan Vega3 scanning electron microscope (Tescan, Brno, Czech Republic).

### 4.8. Semi-Quantitative Assessment of Acute Inflammation in the Urinary Bladder Wall

#### 4.8.1. Measurement of the Relative Thickness of the Lamina Propria

To assess the presence and extent of edema, three H&E-stained cross-sections of the urinary bladder per animal (n = 3) for each time point of the regeneration period were recorded with a Nikon SMZ 800 stereomicroscope (Nikon, Amsterdam, The Netherlands), and the images were processed in the ImageJ program (National Institutes of Health, Laboratory for Optical and Computational Instrumentation, University of Wisconsin, Madison, WI, USA). In each tissue section, the surface area (S1) of the lamina propria and the surface area (S2) of the entire urinary bladder wall were measured, and the relative thickness of the lamina propria (rTh) in the bladder wall was calculated according to the equation:

(2) $$\mathrm{rTh}\;\left(\mathrm{lamina}\;\mathrm{propria}\right)=\frac{S1\;\left(\mathrm{lamina}\;\mathrm{propria}\right)}{S2\;\left(\mathrm{bladder}\;\mathrm{wall}\right)}\times 10$$

#### 4.8.2. Analysis of Neutrophil Infiltration

To assess the severity of acute inflammation, the infiltration of neutrophils in the extravascular tissue of the urinary bladder wall was analyzed in tissue sections with previously performed immunolabeling of neutrophil granulocytes. Three immunolabeled tissue sections per animal (n = 3) for each time point of the regeneration period were examined with an AxioImager Z1 fluorescence microscope (Carl Zeiss, Oberkochen, Germany), and a score system was designed according to the abundance of infiltrated neutrophils and their distribution in the urinary bladder wall. The abundance of infiltrated neutrophils (parameter 1) and the distribution of infiltrated neutrophils in the urinary bladder wall (parameter 2) were graded semi-quantitatively by the score values as shown in Table 4. Finally, the values of the two parameters were summed up and a semi-quantitative assessment of the acute inflammation severity in the urinary bladder wall at selected time points was obtained.

### 4.9. RNA Isolation and Quantitative PCR Analysis

Small pieces of urinary bladder excised at different time points (n = 3) during regeneration, frozen at −80 °C, were homogenized in 200 µL of Qiasol lysis buffer with Tissue-lyser II (50 Hz) (Qiagen, Hilden, Germany). The homogenized samples were warmed to room temperature and total RNA was isolated using RNeasy^®^ Plus Universal Mini Kit following the manufacturer’s instructions. The purity and amount of RNA was determined by measuring the optical density (OD) with a NanoDrop 2000c (Thermo Scientific, Waltham, MA, USA) at a ratio of 260 to 280 nm. cDNA was generated from 0.5 µg of total RNA using the Reverse Transcription System (Promega, Madison, WI, USA) with oligo (dT) primers, and RT-PCR was performed in the Thermal cycler (Applied 144 Biosystems, Foster City, CA, USA). 5x HOT FIREPol^®^ EvaGreen^®^ qPCR Mix Plus (ROX) (Solis BioDyne, Tartu, Estonia) with *Cox-2*-specific primers (*Cox-2*-forward 5′-TACCAGTCTCTCAATCAGTA-3′ and *Cox-2*-reverse 5′-TGGTAGGCTGTGGATCTTGCA-3′) and *Saa3*-specific primers (*Saa3*-forward 5′-TGCCATCATTCTTTGCATCTTGA-3′ and *Saa3*-reverse 5′-CCGTGAACTTCTGAACAGCCT-3′) were used for performing quantitative PCR (qPCR) with a StepOne™ Real-Time PCR System (Applied Biosystems, Foster City, CA, USA). Ribosomal protein L3-specific primers (forward 5′-CCTCTGGTGAAGCCCAAGATC-3′ and reverse 5′-TCTGGGTTTCCGCCAGTTT-3′) served as an endogenous control for normalization. All measurements were carried out in duplicate. The melt curves of products showed only one peak in each PCR reaction, and standard curves were performed to determine efficiency, which for both primers was between 85% and 103% with R2 0.98 (*Saa3*) and R2 0.99 (*Cox-2*). The fold change in expression was calculated using the 2^−∆∆CT^ method with normalization to the level of L32.

### 4.10. Statistical Analysis

Data are presented as the median and interquartile range (IQR), except for TEER, which is presented as the mean and standard deviation (SD). A two-tailed unpaired Student’s *t* test (α = 0.05) was applied to evaluate the differences in TEER values between young and old mice at particular time points. For the apoptotic and proliferative index, the relative thickness of the lamina propria (rTh), and gene expression, a Kruskal–Wallis test (α = 0.05) with a post-hoc Dunn’s multiple comparison of each time point after chitosan treatment with a control samples was performed. Graphs were created and analysis performed using Microsoft Excel and the GraphPad Prism software version 7 (GraphPad Software Inc., San Diego, CA, USA).

## Figures and Tables

**Table 1:** Scoring results of neutrophil infiltration in the urinary bladder wall of young and old mice.

	Neutrophil Infiltration			
	Time Points after Chitosan Treatment	Abundance (Score Value 1)	Distribution (Score Value 2)	Sum of Score Values 1 and 2
**Young mice**	Control	0	0	0
	2 h	3.5	2.5	6
	Day 1	3.5	3.5	7
	Day 2	3.5	3.5	7
	Day 5	1	1	2
	Day 10	0.5	0.5	1
**Old mice**	Control	0	0	0
	2 h	2	2.5	4.5
	Day 1	4	2.5	6.5
	Day 2	0.5	1.5	2
	Day 5	0.5	0.5	1
	Day 10	0.5	1.5	2

**Table 2:** Primary and secondary antibodies used for immunohistochemistry on paraffin sections.

Primary Antibodies	Secondary Antibodies
Anti-Ki-67 antigen (1:100; rabbit monoclonal/SP6; Abcam, Cambridge, UK)	Swine anti-rabbit (1:200; biotinylated, Dako, Glostrup, Denmark) or Goat anti-rabbit (1:200; Alexa Fluor^®^ 555; Invitrogen, Carlsbad, CA, USA)
Anti-uroplakins (AUM) (1:10,000; rabbit polyclonal; courtesy of T.T. Sun from New York University, New York, NY, USA)	Goat anti-rabbit (1:400; biotinylated, Dako, Glostrup, Denmark)
Anti-neutrophils (1:100; rat monoclonal/NIMP-R14; Abcam, Cambridge, UK)	Donkey anti-rat (1:300; Alexa Fluor^®^ 488; Invitrogen, Carlsbad, CA, USA)
Anti-active caspase 3 (1:10; rabbit polyclonal; Abcam, Cambridge, UK)	Goat anti-rabbit (1:400; Alexa Fluor^®^ 488; Invitrogen, Carlsbad, CA, USA)

**Table 3:** Primary and secondary antibodies used for immunohistochemistry on cryosections.

Primary Antibodies	Secondary Antibodies
Anti-cytokeratin 20 (1:50; mouse monoclonal/Ks20.8; Dako, Glostrup, Denmark)	Goat anti-mouse (1:200; Alexa Fluor^®^ 488; Invitrogen, Carlsbad, CA, USA)
Anti-neutrophils (1:100; rat monoclonal/NIMP-R14; Abcam, Cambridge, UK)	Donkey anti-rat (1:300; Alexa Fluor^®^ 488; Invitrogen, Carlsbad, CA, USA)
Anti-Ki-67 antigen (1:200; rabbit monoclonal/SP6; Abcam, Cambridge, UK)	Goat anti-rabbit (1:400; Alexa Fluor^®^ 555; Invitrogen, Carlsbad, CA, USA)
Anti-active caspase-3 (1:200; rabbit polyclonal; Abcam, Cambridge, UK)	Goat anti-rabbit (1:400; Alexa Fluor^®^ 488; Invitrogen, Carlsbad, CA, USA)
Anti-collagen IV (1:300; rabbit polyclonal; Abcam, Cambridge, UK)	Goat anti-rabbit (1:400; Alexa Fluor^®^ 555; Invitrogen, Carlsbad, CA, USA)

**Table 4:** Scoring system for semi-quantitative assessment of neutrophil infiltration.

Abundance		Score Value 1	Distribution	Score Value 2
0	0		–	0
≤10	1		Lamina propria	1
10 < *x* < 50	2		Urothelium + lamina propria	2
50–100 Countless	3 4		Urothelium + lamina propria + muscle layer (transmural)	3
Sum of score values 1 and 2: 0–7				

## Abbreviations

AI	apoptotic index
BSA	bovine serum albumin
CH	chitosan
CK20	cytokeratin 20
*Cox-2*	cyclooxygenase 2
DAB	3,3′-diaminobenzidine
DAPI	4′,6-diamidino-2-phenylindole
FCS	fetal calf serum
PBS	Phosphate-buffered saline
PI	proliferative index
rTh	relative thickness of lamina propria
*Saa3*	serum amyloid A3
SEM	scanning electron microscopy
TEER	transepithelial electrical resistance
TEM	transmission electron microscopy
